# Music Morphology Interaction under Artificial Intelligence in Wireless Network Environment

**DOI:** 10.1155/2022/9002093

**Published:** 2022-05-14

**Authors:** LiLan Zhang

**Affiliations:** Art Department, Puyang Vocational and Technical College, Puyang 457000, Henan, China

## Abstract

Music has become the main information carrier, and music and its emotional expression are accurately classified to obtain relevant information. However, how to classify music accurately is a problem that needs to be discussed. The concept and feature extraction strategy of the morphology of music are described. Moreover, the feature extraction and morphological classification elements of digital music are introduced. Next, music morphology is recognized and classified based on the neural network and relief algorithm. In the network, by randomly selecting different music types, the audio data is input into the neural network as the original data and processed by the relief algorithm. The classification and recognition accuracy of the Relief algorithm are verified by changing the number of iterations. The results show that the model's classification accuracy based on the number of iterations is 78.958%. Then, the traditional statistical analysis classification method's performance is compared with the proposed model. The recognition accuracy of the model proposed reaches 92%, which shows that the model can effectively classify music morphology. This study provides a theoretical basis for music morphology recognition in the wireless network environment.

## 1. Introduction

With the development of human-computer interaction (HCI) and multimedia, emotion recognition has become a new research hotspot of artificial intelligence (AI). As an important multimedia material, music is usually analyzed to reveal the emotions it covers, which has attracted extensive attention from relevant researchers. The appreciation and creation of a piece of music cover almost all cognitive processes, such as perception, attention, learning, memory, and emotion. The research on music cognition has great significance to discover the mystery of the brain because music affects people's lives and it has the characteristics of informatization, digitization, networking, and interaction [1]. This brings great challenges to traditional media dissemination. Its special technical nature has attracted more and more concern from scholars [2]. As one of the most widely spread economic industries, digital music can express various emotions and cover a great deal of information, but it does not spread accurately due to immature classification technologies [3]. Therefore, accurately distinguishing the unique morphology of various types of music and spreading it correctly are important tasks [4]. This study provides an automatic recognition technology of music morphology and emotion classification. Music is the imitation of emotional lives, and emotional expression is the most essential feature of music. Among many musical forms, Chinese folk music undoubtedly shows the characteristics of China's thousands of years of history and culture. Using music emotion cognition technology can preserve Chinese national music and promote rapid and effective emotion retrieval, which has great significance to the inheritance, development, and innovation of Chinese national music.

Digital music is the mainstream form of multimedia dissemination and plays an important role in information dissemination on various network platforms. Chalmers et al. found that it is impossible to disintermediate the music recording industry using blockchain technology in their research. They proposed a new classification method by which participants can know about the listening ratio by using digital technology [5]. The study shows that digital music is still irreplaceable. Relevant statistics tell that having more copyright and transmission channels of various types of music can make the platform in a good position in the network environment, like Spotify. Eriksson mentioned that Spotify's playlist based on container technology (the technology for combining, storing, and transmitting music works) helps the normal logistics operation of the music industry and realize the modularity and automation in enhanced control and remote supervision [6]. Music classification is also essential since there are diversified types of music. Sharma et al. argued that neural networks can classify and identify music morphology. Based on classical music, they found a better classification model by comparing several network models [7]. The classification of music morphology needs to extract the features of music works. Melo et al. studied the timbre texture, tone texture, and rhythms of music works, and extracted the features using the topological attributes of audio signals. The local standard deviation of music signals is mapped to the visibility map by neural networks to extract the features of music [8]. Santarcangelo and Wanke achieved a consensus on voice morphology recognization based on psychology, neurology, and musicology, which shows a similarity between perception philosophy and neurophysiology. This gives the illusion that music morphology is basically based on the ideas of creators [9], which further explains the importance of music morphology classification. The research helps solve the copyright legal problems brought by new technologies and promotes the further development of AI.

Therefore, this study aims to explore a monitoring model for emotion and melody recognition and classification of digital music, which can help people create and search music intelligently, and provide unique insights and basis for the intelligent and diversified development of music. After the concept of music morphology is discussed, a model is implemented for classifying and extracting the emotional features of music works. First, the concepts of music morphology and features are introduced, and then the features of digital music are analyzed. Second, neural networks and the relief algorithm are used to classify and calculate music morphology, obtaining the accuracy rate of music morphology recognition. The classification accuracy is judged by comparing the neural network classification method with other classification methods. The innovation of the research method is to combine relief algorithm with BP neural network (BPNN) to identify the emotional expression characteristics of music to obtain a unique music recognition model.

## 2. Materials and Methods

### 2.1. Digital Music Morphology

**Figure 1:**
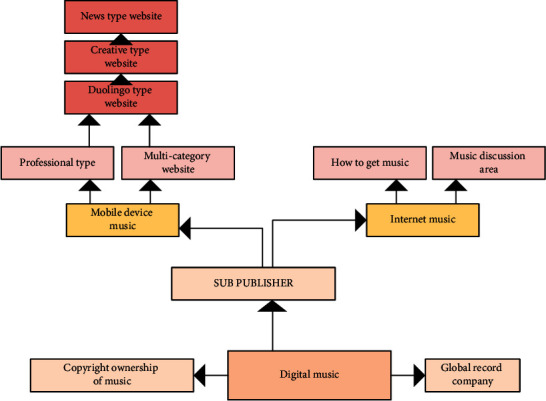
Dissemination direction of digital music.

This section is to describe the communication forms and some special communication methods of popular digital music forms to lead to relevant music concepts. With the progress of science and technology, AI develops rapidly. In terms of copyright, AI has amazing development potential. There are two media of digital music, one is online music [6], which is listened to on the network, and the other is mobile music [10], which can be stored in mobile devices, such as mobile phones, Walkman and other digital devices, and can listen to music anytime and anywhere. These two kinds of music have the same transmission mode. Nearly 70% of the income of digital music comes from online music, and 30% from mobile music. The analysis of the two music forms is helpful to spreading the music works [11], as shown in Figure 1.

**Figure 2:**
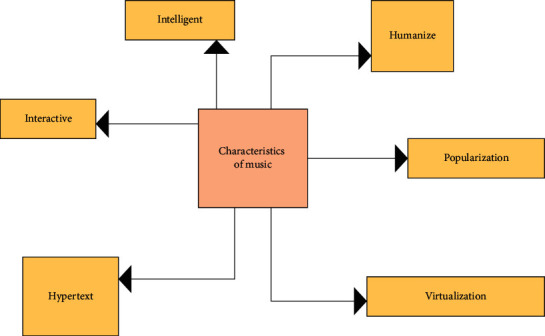
Transmission characteristics of digital music.

Networks and mobile devices are two media based on the computer Internet and mobile phone network. The inevitability that digital music can be widely spread in these two media is discussed [12]. Compared with electronic media, digital media has some unique characteristics, like TV broadcasting, due to the difference in its control technology system [13]. The first two basic characteristics of digital music on the Internet and mobile phones are determined by the following conditions: the characteristics of the media, and networking and dissemination functions of the Internet and mobile phones [14].  Figure 2 shows several transmission characteristics of music.

The characteristics of streaming media are that it has strong immediacy and interactivity. In the study of music communication, it is not difficult to find that model music APP is integrated with social media, so that music producers can upload music works at the first time and share them with their fans and music lovers. Meanwhile, it also allows music audiences to communicate with other music lovers in music software. The platform's use of big data permeates all aspects of users' use. Modern scientific and technological means with strong intelligence can classify different music types such as pop music, ancient style, jazz, hip-hop, and classical music. Besides, they accurately locate the types of users at different levels, aspects, and hobbies according to the big data of users' habits. Differentiation of audience orientation in music communication provides music lovers with a fine music library with personal characteristics. It pushes their favorite types of songs, song lists, and singers for users every day.

### 2.2. Emotional Characteristics of Digital Music

This section will focus on expressing the emotional characteristics of digital music. Each music has its unique emotional expression characteristics. Therefore, different musical emotional expressions will be introduced. Music cognition research is interdisciplinary and has become an international research hotspot and attracted more and more attention. From a cross-cultural perspective, it is a new topic to reveal the impact of music on cognition. The analysis and extraction of music features is the basis for the classification of folk music. The selected features should contain as much feature information as possible, such as typical time-domain and frequency-domain features [15]. Before the classification of digital music is discussed, some music signal processing technologies of feature extraction methods are introduced in detail, gaining the audio data set, and the music feature vector of folk music classification [16].  Table 1 shows the emotional characteristic of music.

**Figure 3:**
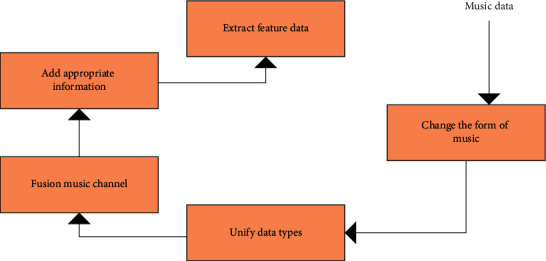
Feature extraction of folk music.

Generally, the technology used for music feature extraction is called short-time processing technology, so it is necessary to define audio structural units with larger time granularity (usually several orders of magnitude longer than the frame) based on the frame, which is called audio segments [17]. The audio segment consists of several frames with a period, such as the voice audio segment and music audio segment. The characteristics of audio segments are calculated based on the characteristics of audio frames. The information that cannot be obtained from the audio frame can be extracted from the audio segment. Here, folk music is discussed as an example. When music features are extracted through short-term processing, pre-processing must be carried out first, and features are achieved through a series of processes [18].  Figure 3 shows the feature extraction process of folk music.

Digital music has several basic characteristics, as shown in Table 2. The characteristics cannot be regarded as the unique features in the classification, but they are the premise on which music features are identified.

Music emotion classification is an interdisciplinary research topic that mainly involves psychology, auditory physiology, musicology, signal and information processing, and pattern recognition. Different from the classification basis of music such as language, style and genre, listeners have great subjectivity in their emotional understanding and definition of music. Using intelligent information processing methods by computers to replace manual labeling methods to classify, manage, and retrieve music has become a research hotspot of music emotion classification. In related technologies, the machine learning algorithm is used to classify music emotion. However, because the training and application of the algorithm model depend on complete music or songs, the learning speed of the algorithm is slow, the classification speed is low, and the accuracy of classification is affected. Technical implementation elements are to overcome the problem of using machine learning algorithms to classify music emotion in related technologies to a certain extent.

### 2.3. Dissemination Modes of Digital Music

**Figure 4:**
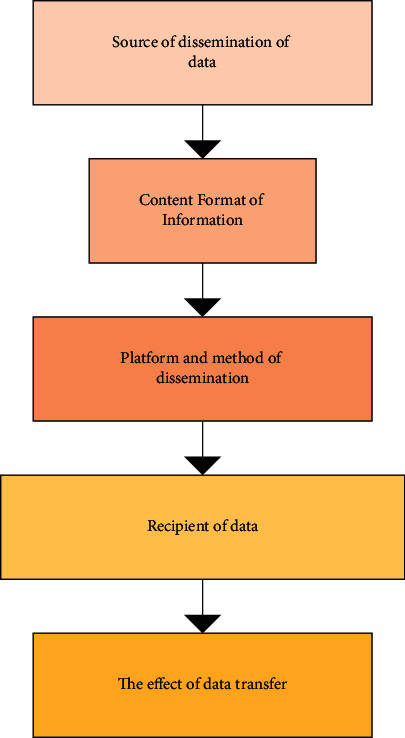
Dissemination structure of the Laswell mode.

The transmission mode of digital music generally varies with the centralized network platform of music. However, most music adopts the Laswell mode as the communication mode, so this section is to briefly introduce this mode. The communication platform based on new media lays the foundation for the development of the design of dynamic digital images, which will have a positive impact on the communication of local customs. The image that changes shapes with the flow of time is designed and stored by digital means. This is the most popular way because of the intuitive and easy structure, fast communication, and audio-visual characteristics. The transmission mode of digital music has attracted the attention of many researchers. At present, the dissemination mode follows the Laswell mode [19]. This mode shows the dissemination scope and content. There is an independent study on each dissemination link.  Figure 4 shows the dissemination structure of the Laswell mode.

The Laswell mode almost covers the main links in the dissemination process, but it lacks a link: feedback. In the feedback link, a part of the output of a system returns to the input end of the system. The advantage of the feedback link is that it can better adjust the output of the system. The Laswell mode also has some limitations. For example, the unidirectional dissemination mode ignores the feedback effect of the audience on information, and the audiences just receive the information in the dissemination process; there is no feedback mechanism, and the audiences have no opportunity to become the main body in dissemination [20]. This transmission mode takes the satisfaction of the communicator rather than the audience as the measuring standard of music. Laswell's feedback connection mode is the linear mode, that is, the flow of information is linear and unidirectional. This model clearly summarizes human communication activities into a process composed of five links and elements, which is a great innovation in the history of communication research, and provides a specific starting point for the later study of the structure and characteristics of the mass communication process. The five main research fields of mass communication, namely “control research,” “content analysis,” “media research,” “audience study,” and “effect analysis,” are also developed from this model. However, it fails to pay attention to the feedback element and ignores the two-way nature of communication.

### 2.4. Relief Algorithm Based on BPNN

**Figure 5:**
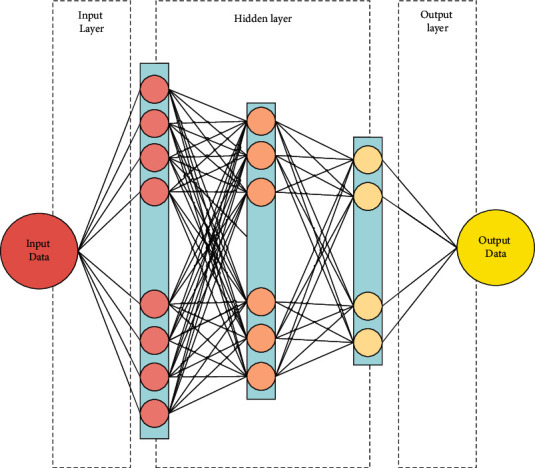
Structure of neural networks.

The music recognition model proposed takes BPNN as the technical carrier and relief algorithm as the data processing means. The specific calculation process of the model will be explained here. The back propagation (BP) model [21] transforms the input F02D output problem of a group of samples into a nonlinear optimization problem. BPNN uses the optimization algorithm (gradient descent method) to obtain the weight by iterative operation. In terms of learning and memory problems, it adds a hidden layer to adjust the parameters to obtain the optimal solution. The number of hidden nodes of BPNN is based on experience and the results of many experiments. The newly added samples should affect the learned samples, and the number of characteristics of each sample must be the same.  Figure 5 shows the structure of neural networks.

Input gate *i*_*t*_ of neural networks and state *C*_*t*_^∼^ after data processing are important parameters [22]. Output is used to update output *C*_*t*_^∼^, and the equations are

(1) $$\begin{array}{c} i_{t}=\delta \left(W_{xi}x_{t}+W_{hi}h_{t-1}+b_{i}\right),\\ C_{t}^{∼}=\tanh\left(W_{xC}x_{t}+W_{hc}h_{t-1}+b_{c}\right). \end{array}$$

*W*_*x*_ is the weight value of the input data of the neural network. *W*_*h*_ is the weight value of the *t*-th input data from the neuron. *h*_*t*−1_ is the output data of the previous neuron. *b* is the deviation corresponding to the neuron.

Relief (Relevant Features) is a famous filtering feature selection method. Relief is a series of algorithms, including the first proposed Relief and later extended Relief-F and RRelief-F. Among them, the RRelief-F algorithm can solve the multi-classification problem, and it aims at the regression problem where the target attribute is continuous value. The earliest proposed relief algorithm is mainly aimed at the binary classification problem. This method designs a “correlation statistic” to measure the importance of features. The statistic is a vector, and each component of the vector is the evaluation value of one of the initial features. The importance of the feature subset is the sum of the relevant statistics corresponding to each feature in the subset. It reveals that this “correlation statistic” can also be regarded as the “weight” of each feature. A threshold can be specified. Then, it needs to select the eigenvalue corresponding to the correlation statistic larger than the threshold. Also, it can specify the number of features expected, and then choose the number of features with the largest measurement component of the correlation statistic.

Relief [23] originally solves two classification problems. Later, it is improved and can replace a series of algorithms. It can evaluate features according to their ability to distinguish similar samples. Its core idea is that good features should make similar samples close and different samples far away.

Relief is recognized as a filtering feature selection method. It helps solve many complicated problems. The algorithm divides the two classification problems into multiple classes and gives the processing methods. It can also be used in solving regression problems. The equation of correlation matrix *R* of the solution sample is

(2) $$\begin{array}{c} \left|R-\lambda I_{p}\right|=0. \end{array}$$

*ρ* characteristic roots are obtained by equation (2), and the confidence of their principal components is obtained by

(3) $$\begin{array}{c} \frac{\sum_{j=1}^{m}\lambda_{j}}{\sum_{j=1}^{\rho }\lambda_{j}}\geq C,\;0＜C＜1. \end{array}$$

In equation (3), *C* is the ratio of the amount of information to be retained [24], namely the utilization rate of information, which is usually a man-made constant. When *j* = 1, 2,…, *n* in *λ*_*j*_, Rb = *λ*_*j*_*b*  and *b*_*j*_^*o*^ can be obtained by solving the equation. And then the standardized index variables are converted into main components by

(4) $$\begin{array}{c} U_{ij}=Z_{i}^{T}b_{j}^{o},\;j=1,2,\ldots ,m. \end{array}$$

**Figure 6:**
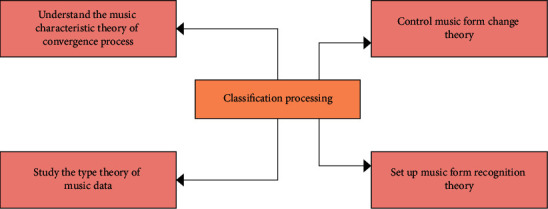
Structure of classification processing.

In equation (5), *U*_1_ is the main component of the first, *U*_2_ is the second, and *U*_*n*_ is the *n*-th. One of the common theoretical foundations of existing machine learning methods is statistics.  Figure 6 shows the classification processing using a classification model.

**Figure 7:**
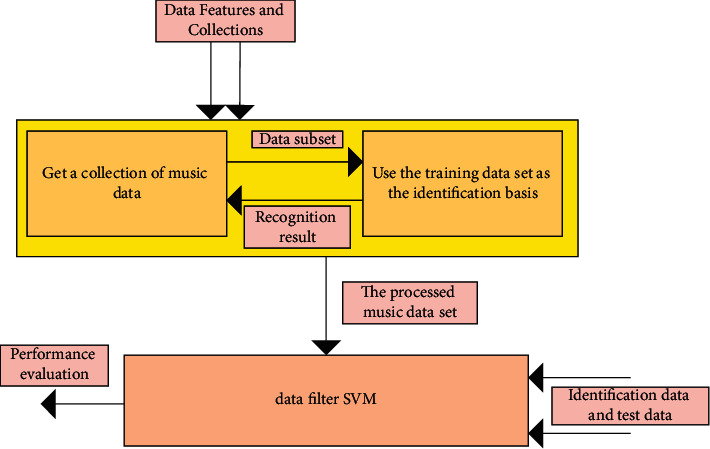
Music recognition model structure of relief algorithm.

Statistics is the most basic analysis method without prior knowledge. However, the traditional statistical pattern recognition methods are based on large samples. The performance of the proposed methods can only be guaranteed theoretically when the size of samples tends to infinity. In practical applications, the size of samples is small, which makes it difficult to achieve good results. Therefore, Figure 7 is the structure of the music recognition model based on the relief algorithm.

The project is to create works of art using artificial intelligence technology. The so-called deep neural network of artificial intelligence is a complex mathematical system that uses big data analysis to learn specific behavior. For example, it includes learning how to identify bicycles by looking for their common models and styles in millions of bicycle photos. It is the principle applied by social software to recognize faces in online photos. Android phones can recognize oral instructions. The translation function of Microsoft's communication software also depends on this technology. Meanwhile, these complex systems can also create art. For example, by analyzing a group of songs, they can learn how to make similar sounds. With this abstraction, deep learning technology, especially content generation technology, can be applied to the creation of pop music. In fact, music is not just art, and it also includes logic and rules, which are good at deep learning. If there is enough data, model capacity and computing power, deep learning can produce better results. Therefore, the above process can correspond to typical deep learning application tasks. The generation of music score and performance skills can correspond to the language generation in natural language processing, because they are symbolic representation, while sound generation can correspond to speech synthesis, so that people can learn from the deep learning technology in these mature fields to help music generation.

### 2.5. Support Vector Machine (SVM)

As a data classifier, SVM can integrate the data processed by neural network for result data analysis. SVM [25] is a secondary classification model. Its basic model is defined as the linear classifier with the largest interval in the feature space. Its learning strategy is to maximize the interval, which transforms the problem into a convex quadratic programming problem. Some data points in different classes are given, a linear classifier is needed to divide these data into two classes. If *x* is used to represent data points and *y* represents categories (*y* can take 1 or −1, representing two different classes respectively), the learning goal of a linear classifier is to find a hyperplane in the *n*dimensional data space. The equation is

(5) $$\begin{array}{c} g\left(z\right)=\frac{1}{1+e^{-z}}. \end{array}$$

Regression is to learn a 0/1 Classification Model from features, which takes the linear combination of features as independent variables. Because the value range of independent variables is from negative infinity to positive infinity, the logistic function (or sigmoid function) is used to map the independent variable to (0, 1), and the mapped value shows that it belongs to the probability of *y* = 1.

At present, music development faces the high professionalism of music creation and the low personalization of consumer music. As works of a certain style occupy the music market, the wind of imitation blows instantly, and the individuation of music is gradually oppressed and limited. In this context, the creation of music by artificial intelligence can bring new solutions. Once artificial intelligence has learned more about how to write music through learning and training, this ability can be given to more people, so that everyone can become a music creator. Meanwhile, artificial intelligence can create personalized music according to the training content, which greatly enriches the types and styles of music.

### 2.6. Music Data Selection for Experiment

Different music is randomly selected from the network in the result verification link. The developed music recognition model is used on the music platform, and the famous dance songs such as The Blue Danube, Swan Lake, Turkish March, and Schubert Serenade are selected for experiments. 10 second segments are randomly selected from the above songs. The design in the recognition program library can basically express the corresponding emotion. Each dance music corresponds to a change of action, either cheerful, lyrical, or passionate. The experimental analysis results are as follows.

## 3. Results and Discussion

### 3.1. Accuracy Analysis of Music Morphology Classification Based on Relief

**Figure 8:**
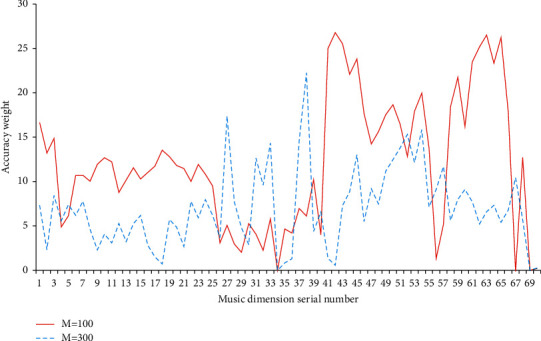
Classification results of Relief.

In this link, Relief based on neural networks is used to test the classification of folk music. According to two different iteration times, the first iteration times are 100 and the second times are 300. The classification accuracy of music morphology under the two classification strategies is tested. The abscissa in the coordinate is the dimension serial number, and the ordinate represents the weight of the classification accuracy. The statistical results are shown in Figure 8.

Figure 8 shows that the iteration times in the experiment have little impact on the accuracy. In the 34-th and 69-th music dimensions, the weight of these two accuracy rates is 0, and the other weight values are greater than 0. Therefore, the data in 34 and 69 dimensions have no impact on the accuracy analysis and can be deleted. The research data are obtained by using a neural network as the carrier of Relief algorithm. The SVM classifier mentioned above is used to calculate the probability classification of data respectively. The overall data processing results reveal that the average recognition accuracy is 78.958%. This accuracy rate shows that Relief can classify music morphology more effectively and accurately.

In addition, in the feature recognition of all dimensions, Relief can also ensure accuracy even if the weight value is small, indicating that weights have little impact on music morphology classification. This shows that the neural network model based on Relief can recognize the music morphology more accurately. The results show that the model can accurately identify the melody, lyrics, and emotional expression of folk music, which proves the good recognition performance of the model.

### 3.2. Accuracy of Identifying Music Morphological Features Using the Neural Network Model

**Figure 9:**
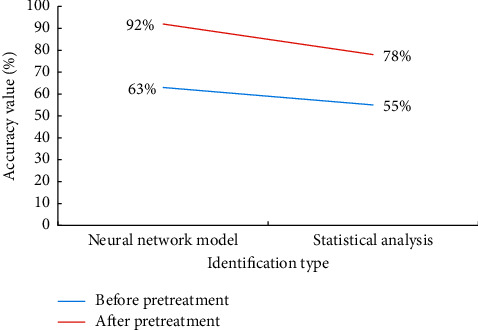
Accuracy rates of music morphology recognization.

Based on the experiment, Relief is used to identify the music morphological features, and the accuracy is compared with the traditional statistical classification and recognization methods. The situations in the experiment are analyzed: the first is before the data preprocessing of music feature extraction, and the second is after the data preprocessing of music feature. The accuracy rates of music morphology recognization in two situations are discussed to judge the practicality of the model in music morphology recognization. The results are shown in Figure 9.

The data curve in Figure 9 shows that two models are used for music morphology recognition before preprocessing various characteristic data of music. The accuracy of the traditional statistical recognition rate is 55%, while the accuracy of Relief recognition model is 63%, which is 8% higher than that of conventional methods. After preprocessing the music data, the accuracy of the traditional recognition method is improved to 78%, while the recognition rate of Relief model increases greatly, which is 92%, 14% higher than that of traditional methods. It can prove that the recognition accuracy of Relief model for melody, lyrics and emotion of folk music is better than that of conventional statistical recognition methods. Generally speaking, there are some deviations in the data, and the data points expressed by various features may cross in the vector space. Since the projection process of the data is a linear change, Relief can make the accuracy rate improved by data fusion. From fault tolerance, the error in the data has an impact on the classification performance of the algorithm, but it has little impact on neural networks. This is because the neurons in the neural network are independent, which reduces errors in the network structure. Therefore, the music platform of Xingtai City is taken as the sample, and neural networks are used to classify its music morphology. Neural networks have a good adaptive ability. It can classify scattered and unorganized data independently without following any rules. Compared with other classification and recognization methods, neural networks use fuzzy logic to imitate human thinking mode and meet the technical requirements of artificial intelligence (AI), because they have stronger adaptability in unknown situations.

## 4. Discussion

The emotional expression connotation and emotional characteristics of music are studied. How to identify and classify the form of music is explored. By expounding the concept of music intelligent production, an intelligent model which can recognize and classify music forms is established based on BPNN, Relief algorithm, and SVM classifier. The model's performance verification results show that the model's average accuracy is 78.958%. Moreover, after preprocessing the music feature data, the accuracy of statistical analysis is 78%, while the recognition accuracy of the neural network model is as high as 92%. Subsequently, the comparison results show that Baró et al. proposed a complete handwritten music recognition system based on convolution recurrent neural network, data enhancement, and transfer learning, which can be used as the baseline of the research community [26]. The results are similar to the results obtained here, proving that intelligent technology plays an accurate role in music morphology recognition and further verifying the correctness of the conclusion obtained here.

## 5. Conclusion

With the development of network technology, music is used as a carrier to convey different emotions in information communication. Identifying and analyzing emotions in various music types have become a new research topic. The concept and characteristics of music morphology are introduced, and the characteristic elements of digital music are discussed. The spread of digital music is based on the network platform, and the spread technology determines the spread effect of music. Neural networks and affective computing are hot spots in artificial intelligence. Therefore, Relief and neural networks are used to build the music morphology classification and recognition model. Moreover, the probability calculation strategy of the SVM classifier is used to analyze the classification and recognition performance of the model. Relief's classification and recognition accuracy are verified by changing the number of iterations. It is found that the number of iterations has little effect on the classification accuracy of the model, and the average recognition accuracy of the model is 78.958%. On this basis, the neural network model is compared with the traditional statistical classification methods to explore the two states before and after the preprocessing of music feature data. Numerical comparison shows that the recognition accuracy of the neural network model is 92%, which is higher than the traditional statistical classification strategy. Therefore, the music recognition model based on neural network and Relief can effectively recognize the music form with high accuracy and good performance. The disadvantage is that there is not enough comparison between different classification algorithms. In the follow-up work, other algorithms will be introduced and compared.

## Figures and Tables

**Table 1:** Emotional characteristics of music.

Characteristics	Emotional characteristics
Subjective characteristics	Music works express the feelings of reality through the author's mind, which shows their subjectivity.
Hierarchical features	People's feelings, the music style, and the expressed emotion are the cognitive methods of music. Music can be transformed into people's inner feelings.
Objective characteristics	Emotion exists in people's hearts. It can be expressed with the art form with the same emotional structure.
Fuzziness feature	Emotion is implicit knowledge that can be deconstructed through symbols and artistic forms. Music can be viewed as the corresponding relationship between artistic symbols and emotions.
Integrity characteristics	In music creation, the emotion cannot be expressed by a single independent music symbol. If he perceives the rhythms or beats only, the creator cannot feel the emotions in it.

**Table 2:** Basic characteristics of music.

Basic characteristics of music	Expression forms of characteristics
The frequency spectrum of energy	The frequency spectrum of energy is a way to convert music signals into the frequency domain and then obtain frequency energy. Fourier transform the information of each frame is performed.
The frequency spectrum of musical amplitude	The frequency spectrum of music amplitude is similar to that of the energy frequency spectrum, but their calculation processes are different.
Square diagram of music rhythms	The square diagram of rhythms represents the content of music clips.

## Data Availability

The simulation data used to support the findings of this study are included within the article.
